# Age-Related Variance in Performance versus Ratings of Attention and Impulse Regulation in Children: Implications for the Assessment of ADHD

**DOI:** 10.3390/brainsci12081033

**Published:** 2022-08-04

**Authors:** Rachael E. Lyon, Jala Rizeq, David B. Flora, Rhonda Martinussen, Brendan F. Andrade, Maggie E. Toplak

**Affiliations:** 1 Department of Psychology, York University, Toronto, ON M3J 1P3, Canada; 2 LaMarsh Centre for Child and Youth Research, York University, Toronto, ON M3J 1P3, Canada; 3 School of Health & Wellbeing, University of Glasgow, Glasgow G12 8QQ, UK; 4 Department of Applied Psychology and Human Development, Ontario Institute for Studies in Education (OISE), Toronto, ON M5S 1V6, Canada; 5 Child Youth & Emerging Adult Program, Centre for Addiction and Mental Health, Toronto, ON M6J 1H4, Canada; 6 Department of Psychiatry, University of Toronto, Toronto, ON M5S 1A1, Canada

**Keywords:** self-regulation, executive function, behavior ratings, longitudinal, attention-deficit hyperactivity disorder (ADHD), strengths and weaknesses of ADHD symptoms and normal behaviors rating scale (SWAN)

## Abstract

Executive function task (EF) deficits are hypothesized to underlie difficulties with self-regulation. However, tasks assessing EF impairments have only been weakly correlated with rating scales that index self-regulation difficulties. A community sample of children and youth aged between 8 and 20 years old were assessed longitudinally. Growth curve analyses and correlations were conducted to better understand how these two types of measures relate to one another across development, as well as the impact of age-related variance. EF was assessed using the Stroop Task and Trail Making test and behavioral ratings of self-regulation were captured using the SWAN scale. EF task performance improved steeply until age 14–15, whereas the SWAN Scale showed small age-related decreases. EF task performance was moderately correlated with age among 8–13-year-olds and to a lesser extent among 14–20-year-olds. SWAN scores were not significantly related to age in either group. Correlations were similar in an ADHD “at-risk” subgroup. EF task performance and parent ratings of attention regulation have different developmental trajectories, which may partly explain why correlations are low to modest in these samples. In particular, age-related variance is an important methodological consideration with significant implications for the assessment of self-regulation in children and youth with ADHD.

## 1. Introduction

The development of self-regulation is characterized in most models and taxonomies as a process whereby an individual acquires the ability to control behavior volitionally in the service of goals or situational expectations [1,2]. The capacity for self-regulation develops consistently across childhood, and there is accumulating evidence that it continues to develop at the cognitive [2], behavioral [3,4] and neurobiological levels [5,6] well into adolescence. Executive function (EF) includes processes such as attention, working memory, planning/organizing and response inhibition, which are neurocognitive processes integral to self-regulation and daily functioning [7,8]. EF deficits are hypothesized to underlie difficulties with attention and impulsivity, behavior characteristic of neurodevelopmental disorders (NDDs), such as attention-deficit hyperactivity disorder (ADHD) and autism spectrum disorder (ASD) [9,10,11]. Despite EF’s integral role in NDDs and in the development of self-regulation generally, there are weak correlations between EF tasks and rating scales that index these types of difficulties [12,13,14,15]. To better understand the overlap and divergence among these two types of measures, the purpose of this study was to examine EF task performance and parent ratings of attention and behavior regulation in a community sample of children and youth who were followed longitudinally on three occasions. Developmental trajectories of these measures and correlations among them were examined to better understand whether EF task performance and parent ratings of attention and behavior regulation capture similar age-related variance. On the other hand, if these two sets of measures show differing developmental trajectories, it may explain the divergence reported in the literature.

### 1.1. Executive Function Task Performance

EFs represent a number of top-down neurocognitive processes required for goal-directed behavior [16,17]. These processes are important aspects of cognitive development that predict behavior in everyday life [8,18]. As these neurocognitive processes develop with age, children become increasingly competent in approaching problems, planning and organizing thoughts and behavior, maintaining goals in mind and acting on them and self-evaluation [19,20]. The past two decades of research support a model with three correlated but distinct EFs, namely, inhibition, set-shifting and working memory [21,22,23]. Inhibition or inhibitory control refers to the ability to control attention, thought and behavior in the presence of interfering internal or external stimuli, to overcome automatic impulses and respond appropriately so that with increasing inhibitory control, one is able to better restrict and regulate impulsive behaviors [2,21]. Set-shifting, also known as cognitive flexibility, describes one’s ability to mentally shift from one task to another, utilizing alternative strategies and processing more than one source of information [20]. Updating and monitoring of working memory representations, or simply updating, is a working memory operation that requires replacing old information with new information relevant to the task at hand [24], which is needed to hold out-of-sight information in mind, manipulate it and work with it to achieve goals and meet task demands [25,26,27].

EF skills develop rapidly in the preschool years; however, performance has been reported to continue into late adolescence and peak in early adulthood [28,29,30,31,32]. The developmental trajectory for the maturation of EF depends on prefrontal cortex engagement, particularly the dorsolateral region, to perform these high-level cognitive processes which are not considered fully developed until early adulthood [33,34,35,36]. Both speed and accuracy of inhibitory control continue to mature into adolescence [2]. Cognitive skills underlying the different facets of EF develop at different times. For example, the ability to delay a response (a skill strongly associated with the successful development of inhibitory control) appears to develop earlier than other EF skills. Set-shifting is the last of the three core EFs to emerge (around 7 to 9 years of age), and is thought to build on inhibition and working memory abilities [2].

### 1.2. Behavioral Ratings of Attention and Impulse Regulation

The ability to regulate one’s behavior, impulse and attention is dependent on numerous underlying cognitive skills, such as EFs, that develop with age. The assessment of self-regulation skills in children has been frequently indexed by informant ratings of children’s functioning relative to peers their age (e.g., parent ratings or teacher ratings). Informant-based scales indexing self-regulation have typically focused on attention, hyperactivity and impulse regulation, including items relating to the observed cognitive, motor and impulse control of the child [37]. For example, behavior rating scales of this nature have been used to assess self-control longitudinally [38]. These scales are typically completed by parent or teacher informants, as child self-report is not usually considered reliable for assessing these behaviors [39,40].

Historically, we have been most interested in capturing deficits in these domains due to their role in developmental psychopathology and most clinical scales have been designed to do so. Nonetheless, attention and behavior regulation are critical aspects of healthy development and as such have been examined in community samples as well. For example, the Strengths and Weaknesses of ADHD Symptoms and Normal Behavior rating scale (SWAN) [41], used in this study, allows the assessment of a child’s ability to regulate attention and hyperactivity/impulsivity along the full dimension [42]. The SWAN also differs from most behavior rating scales of this type as the items are worded using a competency-based rather than a weakness-based formulation as in the DSM-5 [43] and has been studied extensively in community samples [42,44].

Longitudinally, parental ratings of attention, hyperactivity and impulsivity problems in a general population sample demonstrated decreasing hyperactivity with age and relative stability of inattention symptoms from early childhood through to late adolescence [45]. Developmental effects obtained by parent ratings on the SWAN and another ADHD scale using a non-clinical sample of 528 pairs of same-sex twins aged 6 to 9 and 488 pairs aged 12 to 20 years of age showed a similar effect [46]. In this cross-sectional study, younger children had more parent-reported problems than the older children on the SWAN for both inattention and hyperactivity–impulsivity subscales [46].

### 1.3. Comparing Executive Task Performance and Parent Ratings of Attention and Behavior Regulation

Two main classes or types of measures have been used to index the development of self-regulation: (a) self- and informant-report questionnaires of behavior observed in real-world settings and (b) performance-based measures, such as executive function tasks [47,48,49]. These classes parallel the performance-based versus rating scale distinction of EF discussed previously in the literature [13,14,15]. Specifically, performance-based measures involve standardized procedures administered by an examiner and usually assess accuracy or response time. Rating measures of self-regulation involve an informant retroactively reporting on the frequency or severity of an individual’s difficulties carrying out everyday tasks and their behaviors related to self-regulation. It is also important to note here that the association between ratings of ADHD severity are significantly correlated with executive function ratings, ranging from *r* = 0.68 to 0.91 [50]. While both types of measures assess the aspects of self-regulation, there is accumulating evidence that they are also conceptually and operationally different [13,14,15]. Specifically, performance-based measures of EF capture optimal performance situations because the parameters for task completion are determined externally by the examiner and are not left up to the participant. In contrast, on rating measures, participants estimate the frequency and typicality of how well they perform in day-to-day situations that are likely to engage executive processes. Their responses are not constrained by an external examiner and there are no explicit instructions to maximize or optimize their ratings. Interpretation of the task is left up to the rater, who must decide on instances from their everyday lives that map onto the questions asked.

Age differences, or development, is another important factor to consider when trying to understand why correlations between these sets of tasks are low to modest. We know that EF and attention and behavior regulation are important skills that develop with age. However, we are yet to examine their developmental trajectories simultaneously. Such an examination would help us better understand the rate of development and how that impacts the association between these two sets of measures.

In this study, we examined whether differences in the rate of development of these skills might explain the low to modest association often reported between these measures. We expected that the performance of EF tasks would improve with age, consistent with the research showing an increase in cognitive abilities over development. We expected parent-reported impulse regulation to improve with age, whereas the ratings of attention were not expected to change with age. Finally, small to modest correlations between these measures were expected within the full sample, as seen in previous large sample studies and meta-analyses [51]. However, we also examined these correlations within different periods of development (8–13 years and 14–20 years) to further examine the effect size of these correlations in these different age groups.

## 2. Materials and Methods

### 2.1. Participants

The current study included data from a sample of children recruited from suburban and rural schools as part of a longitudinal research project. Time 1 and Time 2 data from this study are previously reported [52]. All available data were used. There were 204 children (110 males) at the first measurement occasion (Time 1), with ages ranging from 8 to 14 years old (*M* = 10.15, *SD* = 1.73). Follow-up data were collected twice at three-year intervals. Time 2 includes data from 156 participants (86 males), ranging from 10 to 18 years old (*M* = 13.23, *SD* = 1.84) and Time 3 data were from 134 participants (77 males) from 13 to 20 years old (*M* = 15.97, *SD* = 1.79). The estimated full-scale intelligence score for the sample at the first period of data collection was 108.19 (*SD* = 12.96), based on the Vocabulary and Matrix Reasoning subtests of the WASI (Wechsler, 1999). At Time 2, parents were asked to report their educational attainment. Of the 156 mothers for whom data were available, 48 (23.5%) had professional degrees, 83 (40.7%) completed college or university, 3 (1.5%) had some college or university education, 15 (7.4%) completed high school, 1 (0.5%) did not complete high school and 4 mothers did not report their educational status. Of the fathers, 42 (20.6%) had professional degrees, 67 (32.8%) completed college or university, 14 (6.9%) had some college or university education, 22 (10.8%) completed high school, 3 (1.5%) did not complete high school and 8 fathers did not report their educational status. At both follow-ups, sample retention was good (Time 2: *n* = 156, 76% of the total sample; Time 3: *n* = 135, 66% of the total sample).

### 2.2. Measures

#### 2.2.1. Attention and Impulse Regulation

The SWAN rating scale [41] was used to measure parent ratings of attention and impulse regulation. Parents were asked to rate their child’s behavior relative to same-aged peers for each of the 18 items using a seven-point scale ranging from far below average to far above average. Thus, total scores could range from 18 to 126. The SWAN has been reported to demonstrate good validity and reliability [41,42,44,46,53]. The dependent variable was domain scores on inattention, hyperactivity, impulsivity and an overall SWAN score (total score). A higher score indicated better attention and behavioral regulation.

#### 2.2.2. Executive Function: Inhibition

The Stroop Task [53] was used to measure inhibition. There were three different conditions, each with 24 items arranged in a 4 × 6 matrix: a word reading condition, a color naming condition and an interference condition. The dependent variable of the Stroop Task was the total naming time (in seconds) for the interference condition minus the total naming time for the color condition. Lower scores indicate better inhibition skills.

#### 2.2.3. Executive Function: Set-Shifting

The Trail Making test (TMT) [54,55] was used to measure set shifting. Part A required participants to connect 25 numbered circles in ascending order. Part B required participants to connect 12 lettered and 13 numbered circles, whereby the participant was instructed to alternate between numeric and alphabetic order, going from 1 to A to 2 to B to 3 to C, and so on. Both parts of the test were administered. Total completion time in seconds was recorded for both parts. To remove the effects of individual differences in processing speed, the set-shifting score was obtained by removing the time taken to complete Part A from Part B. Thus, lower scores are indicative of better set-shifting ability.

### 2.3. Procedure

Assessments were administered by trained graduate students and bachelor-level research assistants. Measures used in this study were part of a larger set of questionnaires and tasks administered at each time point. Parent consent and child assent were obtained before starting the study. The administration of task order was as follows: demographics form, WASI Vocabulary, WASI Matrices, Stroop and TMT. One parent completed the SWAN questionnaire for each child.

### 2.4. Data Analysis

The present analyses included data from the two EF tasks and the SWAN scale. There were 13 missing parents’ SWAN ratings at the baseline. Because there was considerable age heterogeneity within each time point, we modeled developmental trajectories of parent-reported attention and impulsivity as a function of age rather than the time point of data collection (i.e., the data are consistent with a cohort-sequential design) [56]. Specifically, because participant ages ranged from 8 to 20 years across Time 1 to Time 3, a long-term developmental trajectory could be approximated by combining the temporally overlapping repeated measures of youth observed at different ages. Thus, with only three time points of data collection, the age-based data were linked to form a common developmental trajectory spanning ages 8 to 20, albeit with substantial amounts of missing data within a given year of age. In fact, the sparseness of data at some ages necessitated collapsing age into the following six categories to facilitate convergence of model estimation: age 8–9 (age category 1; *n* = 90 observations; 49 males, 41 females), 10–11 (age category 2; *n* = 89 observations; 55 males; 34 females), 12–13 (age category 3; *n* = 112 observations; 57 males, 55 females), 14–15 (age category 4; *n* = 107 observations; 59 males; 48 females), 16–17 (age category 5; *n* = 60 observations; 36 males, 24 females) and 18–20 (age category 6; *n* = 28 observations; 13 males, 15 females).

All models were estimated using full information maximum likelihood; this procedure allows data from participants with incomplete data (including longitudinal dropouts) to be incorporated in the model estimation [57], which is essential given that we organized the data according to the age categories described above. All models were estimated using Mplus (version 7.3). Overall, model fit was assessed using the standardized root mean square residual (SRMR) and the root mean square error of approximation (RMSEA), comparative fit index (CFI) and Tucker–Lewis index (TLI) calculated based on the robust chi-square statistic of Yuan and Bentler [58], as implemented by Mplus. For RMSEA and SRMR, values < 0.08 are typically considered indicative of adequate model fit, whereas values of CFI and TLI > 0.90 indicate acceptable model fit.

Relationships among variables were examined within two developmental age ranges, rather than by the time of data collection: childhood (ages 8–13; *n* = 194/182) and adolescence (ages 14–20; *n* = 143/142). These data were analyzed using IBM SPSS (version 27). If participants had more than one observation within the same age range (*n* = 146), scores were averaged across the data points. A total of 92 participants had two observations and 5 participants had three observations in the 8–13-year range, and 49 participants had two observations in the 14–20-year range. An “at-risk” subgroup was identified using an overall SWAN score cut-off at or below the 25th percentile (scores equal to or less than 75). A total of 85 participants were categorized as “at risk” for ADHD. The same correlations were carried out in this subgroup.

## 3. Results

### 3.1. Descriptive Statistics 

Descriptive statistics are presented in Table 1 on each of the raw variable scores. At Time 1, parents reported their children to have well-developed attention and behavior regulation (SWAN total score *M* = 85.74, *SD* = 17.0), where the potential range is from 18 to 126. The 13 children with missing SWAN parent reports did not notably differ from the rest of the sample on the four cognitive measures as these 13 children were within the one standard deviation of the mean of the full sample of children who had no missing data.

### 3.2. Trajectories of Parent-Reported Attention and Impulse Regulation

**Figure 1:**
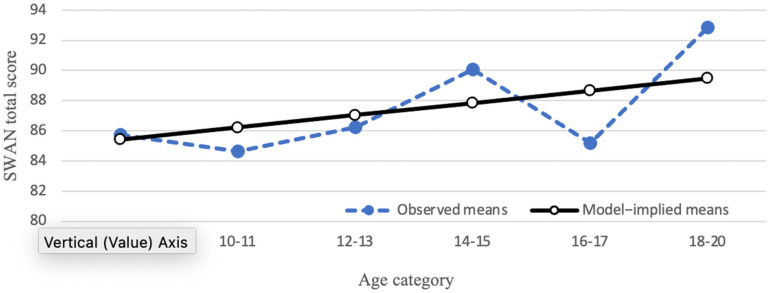
Developmental trajectory of SWAN total score. Closed circles represent observed means; open circles represent model-implied means.

The mean of the SWAN total score displayed a linear trend across the six age categories, such that the means increased from ages 8–9 up to ages 18–20. Impulsivity, hyperactivity and inattention subscales exhibited a similar trend. As such, linear growth curve models were fitted to the data. The linear growth curve model for the SWAN total score is shown in Figure 1. The mean slope was significantly greater than 0 (0.81, *p* = 0.04), while the standard deviation of the slope factor was not significant (2.07, *p* = 0.45). These results suggest that parent-reported attention and impulse regulation show some improvement as children get older, but there are not substantial individual differences in the amount that the SWAN total score changes across age. 

**Figure 2:**
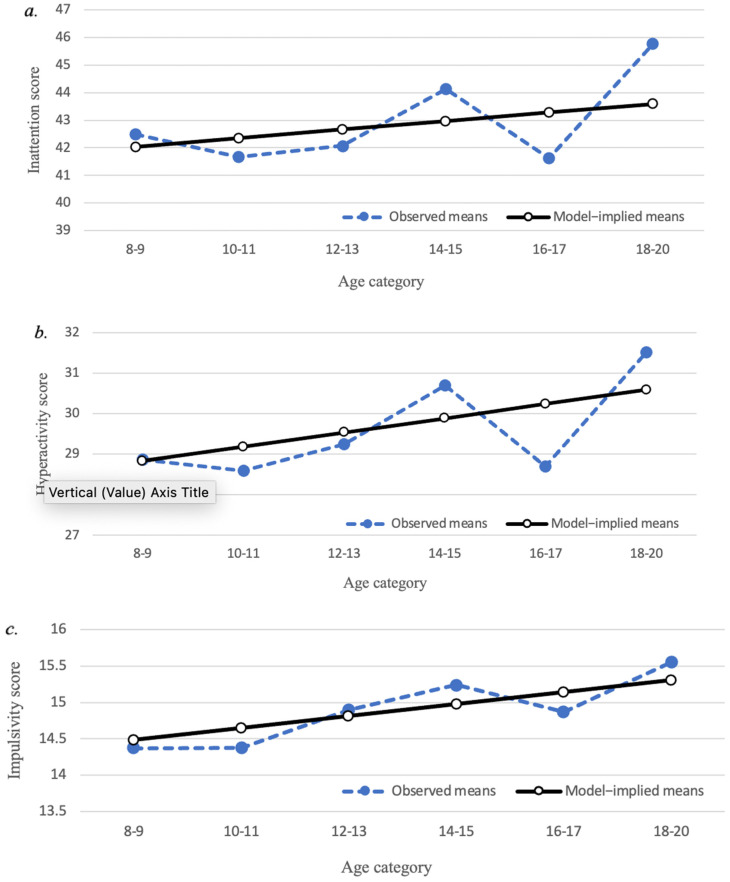
Developmental trajectories of SWAN subscale scores. (**a**) Trajectory of inattention scores; (**b**) Trajectory of hyperactivity scores; (**c**) Trajectory of impulsivity scores. Closed circles represent observed means; open circles represent model-implied means.

Linear growth curve models of the SWAN subscales are illustrated in Figure 2. The mean slopes were significantly greater than 0 for the impulsivity (0.16, *p* = 0.05) and hyperactivity (0.35, *p* = 0.02) subscales, but not the inattention subscale (0.31, *p* = 0.17). Similar to the SWAN total score, the standard deviation of the slope factor was not significant for impulsivity (0.53, *p* = 0.14), hyperactivity (0.17, *p* = 0.96) or inattention (1.29, *p* = 0.38) subscales. Thus, impulsivity and hyperactivity showed some improvement with age, whereas inattention did not. Similar to the SWAN total score, the results suggest that there is essentially no intra-individual heterogeneity in the amount that each of the subscale scores change across age.

### 3.3. Trajectories of EF Tasks

**Figure 3:**
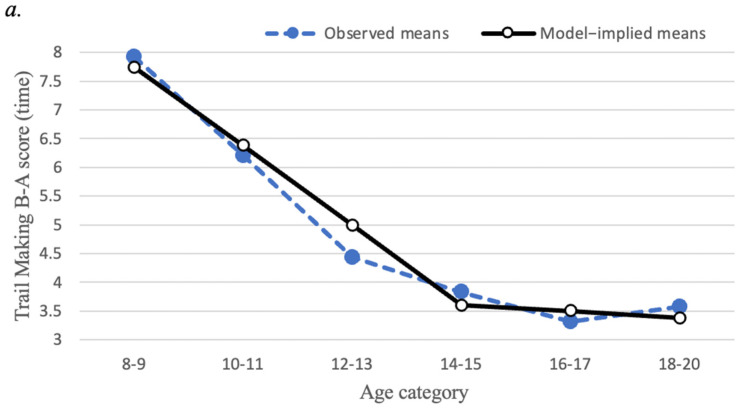

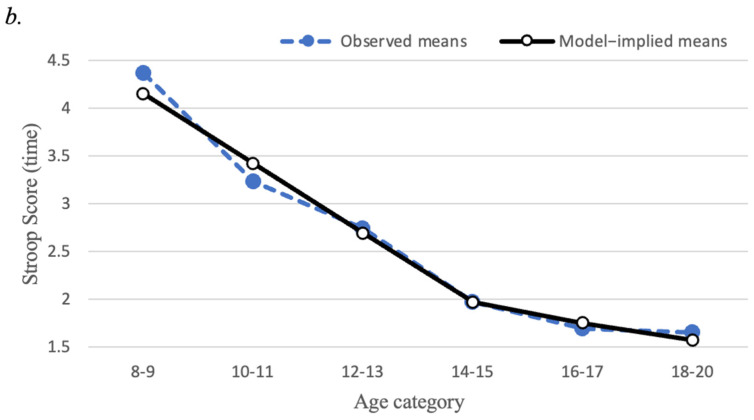
Mean trajectories for executive function task performance (**a**) Trajectory of Trail Making Task Part B minus Part A time; (**b**) Trajectory of Stroop Task interference time. Closed circles represent observed means; open circles represent model-implied means. Score reflects time (in seconds), where less time indicates better performance.

Linear growth curve models of EF measures are presented in Figure 3. The mean scores for each of the EF variables displayed a non-linear pattern across the six age categories described above. Specifically, for set-shifting (Trail Making test) and interference control (Stroop Task), mean scores decreased steadily (reflecting improving performance) up to age 14–15, then showed less steep decreases from ages 14–15 to ages 18–20. To represent this non-linear pattern, a piecewise linear latent growth model was estimated because of its interpretational advantages over alternative models for non-linear growth, such as a quadratic growth model [59]. Specifically, we estimated models with two separate linear segments of time, the first segment representing linear change from ages 8–9 to ages 14–15, and the second representing linear change from ages 14–15 to ages 18–20. Importantly, these models allow the linear slopes to differ across these two time segments, thereby representing the overall non-linear pattern. Furthermore, the models were specified so that the intercept factor represented the level of EF at ages 14–15 rather than the initial timepoint (ages 8–9).

For Trail Making Part B minus Part A time, the first linear slope factor mean of −1.39 (*p* < 0.001) indicated that Trail Making scores decreased steeply from ages 8–9 to ages 14–15. Next, the second linear slope factor mean of −0.14 indicated a less steep, non-significant (*p* = 0.38) average decrease from ages 14–15 to ages 18–20. The standard deviations of the first linear slope factor (*SD* = 1.07, *p* = 0.03) and second linear slope factor (*SD* = 1.09, *p* = 0.04) were both significant, suggesting that there are substantial individual differences in the amount that Trail Making scores change across age.

For the Stroop Task interference time, the growth model converged to a proper solution only after the variance parameters for the two slopes were fixed to zero. The first linear slope factor mean of −0.74 (*p* < 0.001) indicated that Stroop scores decreased steeply from ages 8–9 to 14–15 years of age. Next, the second linear slope factor mean of −0.21 (*p* < 0.001) indicated a less steep average decrease in Stroop scores from ages 14–15 to 18–20 years of age. Because the variances of the two linear slopes were fixed to zero, the model suggests that essentially there is no intra-individual heterogeneity in the amount that Stroop scores change across age.

### 3.4. Correlations

Correlations are reported in Table 2, Table 3 and Table 4. Age displayed a modest correlation with EF task performance in the full sample (Stroop: *r* = −0.63, *p <* 0.05; Trail Making: *r* = −0.55, *p <* 0.05), shown in Table 2. In the full sample, correlations between SWAN scores and EF tasks were mostly small to moderate (Stroop: *r*’s from 0.10 to −0.18; Trail Making: *r*’s from −0.16 to −0.28, *p* < 0.05); all were statistically significant except the relationship between Stroop and SWAN impulsivity (*r* = 0.10, *ns*). Parent rating of attention displayed the highest correlation with the EF tasks, as well as the total SWAN score. Age was not correlated with the SWAN ratings in the full sample (*r*’s from [0.03] to [0.12], *ns*).

Given the steep change in performance of the EF tasks from 8–9 to 12–13 years of age and the far less steep change from 14–15 to 18–20 years of age, correlations between these measures were examined separately in these two different periods of development. In the 8–13-year-old group shown in Table 3, Stroop and Trail Making scores continued to be significantly related to age in the expected direction (*r* = −0.25 and *r* = 0.35, *p <* 0.05), with older children demonstrating better interference and set-shifting than younger children. In the 14–20-year-old group shown in Table 4, the Stroop displayed a smaller significant effect size than with age, compared to the younger age group (*r* = −0.18, *p* < 0.05). The correlation with Trail Making being even smaller and not statistically significant (*r* = −0.16, *ns*). Consistent with the full sample, age was not correlated with parent-reported attention and impulse regulation. As shown in Table 4, age was not significantly correlated with SWAN ratings in the total sample (*r*’s from [0.03] to [0.12], *ns*), nor in childhood (*r’s* from [0.01] to [0.06], *ns*) or adolescence (*r*’s from [<0.01] to [0.03], *ns*).

Finally, the correlations between the EF tasks and SWAN ratings displayed somewhat different patterns in childhood and adolescence, as shown in Table 3 and Table 4. In childhood (8–13 years old), correlations were also small to moderate (Stroop: *r*’s from 0.12 to −0.26; Trail Making: *r*’s from −0.22 to −0.33, *p* < 0.05) and followed the same pattern as in the full sample whereby all relationships were significant except between Stroop and SWAN impulsivity (*r* = 0.12, *ns*). Among adolescents (14–20 years old), correlations between the SWAN scores were smaller and often did not reach statistical significance.

Overall, age was correlated with EF task performance in the 9–13-year-old and full samples, and EF tasks also displayed correlations with the SWAN scale in the 8–13-year-old and full samples However, age displayed a much lower effect size with the EF tasks in the 14–20-year-old sample, consistent with the trajectory analyses. In addition, the effect sizes between the EF tasks and SWAN rating were also very small in the 14–20-year-old sample. This overall pattern suggests that age-related variance likely underlies the correlations between EF tasks and SWAN ratings in the younger group. Given that age-related variance in these EF tasks seems to plateau in the older group, the correlations are much smaller between the EF tasks and SWAN ratings in this group, highlighting the lack of correspondence between EF tasks and the SWAN rating.

These same analyses were conducted separately for the group identified as at risk for ADHD. These correlations are shown in Table 2, Table 3 and Table 4. The findings for the ADHD risk group were parallel to the findings in the full sample, as well as across both age groups. In fact, the effect size correlations between the Trail Making and SWAN ratings were smaller for the full 14–20-year-old group than for the subset at-risk for ADHD, demonstrating this pattern even more clearly than in the full sample.

## 4. Discussion

We evaluated the extent to which EF task performance and parent rating measures capture age-related variance to better understand the divergence in these measures which have been theoretically and conceptually related. Overall, attention and impulse regulation as rated by parents using the SWAN improved from ages 8–9 to ages 18–20; however, the overall trajectory is quite flat and appears to be driven by the hyperactivity and impulsivity subscales. Parent ratings of inattention showed no statistically significant change with age. The pattern of impulsive and hyperactive behaviors improving with age and attention skills remaining constant has been previously demonstrated in the literature [60,61,62,63]. In contrast, both EF task performance measures demonstrate notable improvement with age, particularly from 8–9 to 14–15 years of age relative to 14–15 to 18–20 years of age. This trajectory of rapid age-related improvement across childhood that slows in later adolescence has been well documented [30,64,65].

Based on these results, it is not surprising that age was unrelated to the SWAN total and subscale scores across developmental periods. Conversely, the opposite pattern was seen with age and EF task performance. Age was significantly correlated with the performance of both EF tasks in childhood (8–12-year-olds), and only with the Stroop in adolescents (13–15-year-olds). This is in line with the developmental trajectories of Stroop and Trail Making task performance. Findings were largely consistent in the ADHD risk group.

Regarding associations between these measures, SWAN scores were not consistently significantly correlated with EF performance in the full sample spanning 8 to 20 years of age. This finding aligns with the inconsistent and modest correlations reported between performance-based EF measures and behavioral rating scales in the literature [13,14,15,51,64]. A review of informant reports and performance-based measures of executive function demonstrated that the median correlation was only 0.19 [13]. Similar results were found using a latent EF task performance variable, showing low correlations with both the Behavioral Rating Inventory of Executive Function (*r* = 0.11) and the Early Adolescent Temperament Questionnaire (*r* = 0.21) [15].

Given the steep change in the performance of EF tasks from ages 8–9 to ages 12–13 and the far less steep change from 14–15 to 18–20 years of age, correlations between these measures were then examined separately in these two different periods of development. EF task performance was significantly correlated with the SWAN total score and subscale scores in childhood (8 to 13 years of age), except for impulsivity ratings and performance on the Stroop. In adolescence (14 to 20 years of age), correlations between the SWAN parent ratings and Stroop performance were non-significant. There were significant but small associations between most of the SWAN ratings (all except hyperactivity) and Trail Making performance in this age group. The variability in the size of correlation across developmental periods suggests a fundamental role of age.

One explanation for the small and inconsistent relationship between EF task performance and parent ratings is their differential ability to capture developmental change. We know from prior research that the performance of task-based assessments is highly influenced by age. Moreover, there is evidence that age represents a large portion of the common variance between tasks assessing intellectual abilities and EF tasks in developmental samples. A recent study found that controlling for age eliminated the relationship between EF task performance and intellectual abilities [65]. These findings demonstrate that age-related variance is an important common feature of task-based cognitive ability measures. On the other hand, parent ratings of attention and impulse regulation are a qualitatively different measure that may not capture age-related variance in any manner.

Methodologically, it is important to consider that for SWAN, parents are asked to rate their child’s behavior relative to other children of the same age. When parents rate how well their child can sustain attention, they may say “above average” when the child is eight, but also indicate the same rating at age ten. The instructions for the SWAN scale do not provide any developmental or age-based reference as part of the assessment. The instructions for this scale are very similar to many scales of this type, as shown in Table 5.

In the instructions to raters, many of these scales do not provide any developmental reference point (such as: “rate your child compared to other children of the same age”), and instead ask parents to rate the child’s behavior over a recent period of time (e.g., the last six months). Instead of assessing any age-related changes, these scales focus on deficits or difficulties in attentional and self-regulation skills. Thus, even methodologically, rating scales do not capture age-related variance. One possible direction may be to integrate developmental anchors or to explicitly ask the rater to consider age-related differences based on a particular period of time, such as making a current rating relative to an earlier period for each item. However, even if such instructions were provided, this would still be methodologically flawed as individual raters will likely differ in their personal reference points for what is expected at different periods of development. Overall, the same pattern of findings was obtained for what was defined as an ADHD risk group, based on the bottom 25th percentile of this community sample of children. While this cut-off was based on identifying the children who were least well developed in attention and impulse regulation, this may be considered a limitation of the current study. However, SWAN scores have been used to identify children at risk for ADHD based on more elaborate cut-off metrics [80].

The SWAN does not have published normative data available and to the best of our knowledge, no research has specifically investigated age effects for this scale. One study comparing the SWAN to another ADHD rating scale notes age-related differences, whereby children in the younger age group (6- to 9-year-olds) were rated as more impaired on the SWAN than children in the older age group (12- to 20-year-olds) [46]. However, the largest difference in group mean scores on the inattention and hyperactivity/impulsivity subscales was 0.33, which is comparable to the small changes across developmental periods found in the present study. Several scales of this type do not have age-based norms (see Table 5). However, for those scales in Table 5 that do have age-based norms, it is not clear whether these age-based norms necessarily suggest age-related differences in the item ratings of these scales.

The present results suggest parent ratings of behavior and EF task performance do not converge within and across development as we might expect if they were indeed measuring the same construct. The small and variable relationships between these measures raise important questions about how we define and measure behaviors related to attention and self-regulation. Based on current findings and trends in the literature, we are positing that a differential ability to capture age-related variance may explain, in part, the weak correlations obtained between the EF tasks and the SWAN scale in developmental samples. For the SWAN rating scale, parents are effectively asked to control for age in their ratings by explicitly asking them to compare their child’s behavior to other children the same age. Due to this scale property, they may continue to rate their child as “average” or “below average” despite a change in the frequency of behaviors over time. Alternatively, EF task performance is based on objective indicators, such as accuracy and reaction time, which have been shown to be developmentally sensitive with the performance of these tasks improving steeply throughout childhood and leveling off in adolescence. This trajectory looks very different compared to the parent ratings of behavior.

### 4.1. Considerations for ADHD Assessment

It is important to place these findings into the larger context, including the consideration of theoretical implications and translational applications for the assessment of ADHD. Most explanatory models for ADHD have focused on EF deficits [10], which has led to the understanding of EF as critical to developmental improvement in attention and impulse regulation. Structural and functional brain imaging research supports a relationship between ADHD and EF deficits. Findings suggest that specific areas of the brain that are highly related to executive function processes (e.g., frontostriatal and frontoparietal networks) are underactive in those with ADHD [81]. There is also evidence for delays in cortical maturation [82,83,84] and decreased volume of these regions [85]. However, similar to the behavioral research, findings from neuroimaging and neurocognitive studies that focus on precise neuropsychological deficits and brain regions involved in ADHD are not always consistent. The magnitude, direction, localization, laterality and clinical significance of the functional and structural abnormalities differ from study to study [86,87,88]. In addition to the neural bases of behavior, there is emerging evidence that psychophysiological processes (i.e., anatomical–functional interplay among central and peripheral nervous systems) may play a role in psychiatric conditions [89] which may further explain the lack of consistency in the literature. Indeed, there has been considerable progress and change within the field of ADHD with the accumulation of studies documenting the relationships between these measures and advances in our understanding of the complexity and heterogeneity in the presentation of symptoms among individuals with ADHD [81], as well as the issues related to the diagnostic taxonomies we use [90].

The focus of the current paper was on measurement issues and understanding the implications for the models and assessment of ADHD. The diagnosis of ADHD has been primarily based on criteria from the DSM-5 or ICD-11, which are conventionally assessed using clinical interviews and rating scales [81]. The manner in which we operationalize and measure each symptom/criterion has significant implications for the scientific precision of measuring the underlying processes and mechanisms, but also for the individual in whether they do or do not meet criteria for the diagnosis of ADHD. Specifically, our findings suggest that EFs show developmental effects, but the parent ratings of attention/impulse regulation for the SWAN do not. Consistent with other reviews, the current findings suggest that these two types of measures assess different levels of analysis and they should not be considered as equivalent or interchangeable [14,15,79]. This is similar to the case of EF tasks and EF ratings, where the measures should not be interpreted as parallel or interchangeable, despite both carrying the label “EF” [13].

The performance of EF tasks provides information regarding how well the individual behaves and manages in an optimal and highly structured testing environment with considerable direction and guidance from an examiner. This is consistent with the distinction that has been made in the psychometric literature between optimal or maximal performance situations and typical performance situations [91,92,93,94,95,96,97]. Optimal performance situations include standardized testing situations in which task interpretation is determined by the examiner. Here, the examinee is instructed to maximize performance and often receives feedback to ensure that maximal performance is obtained. The goals and expectations are clearly laid out for the examinee in these testing situations. With age, children demonstrate measurably better performance on these tasks given the growth of cognitive capacities, such as those measured in EF tasks.

Alternatively, typical performance situations are far less constrained and there are no explicit instructions to maximize performance. Often, participants are left to interpret the task and determine for themselves what is required or expected of them. Ratings of EF assess typical performance. In the assessment of child ADHD, it is common for different informants to provide information on how well the child manages in less-structured environments relative to the testing situation, such as a classroom with several other children and in the home setting where there is likely even less structure than in the classroom. These ratings provide an assessment of how well the child executes their goals and manages their behavior without explicit guidance. Both domains are useful and valuable in the assessment of ADHD, but they provide different types of information in the context of a clinical assessment. While this type of performance may also change based on age, such differences are not measured by rating tools.

### 4.2. Limitations and Future Directions

These findings should be viewed with certain limitations in mind. We only assessed two of the three defining EF processes [21] in addition to the SWAN scale; thus, it will be important for future research to replicate the results with other measures. Additionally, our sample was also relatively high functioning, which may impact the variability in the rate of change and our ability to detect different trajectories.

Should researchers and clinicians continue to use behavior ratings and performance-based tasks interchangeably, the lack of age-related variance in behavior ratings must be addressed. One possible solution is to integrate developmental considerations into measures of behavior, such as in the instructions, items or in the rating scale. Task instructions or each item could provide explicit instructions to determine a current rating relative to an earlier period in development. The rating or response scale could also include specific reference to whether the behavior was displayed at the current time relative to an earlier period. However, even if such instructions were provided, this would still be methodologically flawed as individual raters will differ in their personal reference points for what is expected at different periods of development. Thus, individual differences in behavior, but not age-related differences, are assessed on these rating measures.

## 5. Conclusions

This study included an examination of developmental trajectories of EF task performance and parent ratings of attention and impulse regulation in a community sample. Furthermore, we demonstrated how different methods used for measuring self-regulation do not necessarily converge within and across development, and highlighted the challenges associated with assessing the relationship among performance-based tasks and parent-reported measures. The small growth in the ratings of overall attention and impulse regulation as opposed to more rapid growth seen in EF, at least early in development, demonstrated the differential nature of the developmental trajectories of behavioral and cognitive aspects of self-regulation. Age-related differences in cognitive ability tasks, such as EF tasks, have been consistently demonstrated in the literature. This growth in capacity and efficiency of processing with age is expected, for example, a 10-year-old will likely have better inhibitory control and be more accurate in solving complex abstract puzzles than a six-year-old. However, in the case of behavior rating scales, the scores and ratings cannot be expected to track age-related changes. One might expect that there may be some age-related differences in children’s behavior, but methodologically, there is no reason to expect that rating scales will capture any age-related differences. Understanding these different indicators of self-regulation can inform the development of early prevention and targeted treatment strategies. For example, informing educators on what to expect within the classroom and parents on what to expect from their child’s development of regulation over time, especially for at-risk and ADHD populations.

## Figures and Tables

**Table 1:** Descriptive statistics of variables at the start of the study.

Variables	*n*	Mean	SD	Median	Range (Min, Max)	Skewness	Kurtosis
Age	204	10.15	1.73	10.00	8, 14	0.58	−0.61
*Executive Function Tasks*							
Stroop Interference Time	204	36.69	14.03	34.50	9.0, 83.0	0.78	0.75
TMT Part B-A Time	204	69.02	44.33	58.90	−2.0, 256.1	1.54	3.26
*Behavior Ratings of Attention and Impulse Regulation*							
SWAN Inattention	191	42.21	9.26	41.00	15, 60	−0.15	−0.38
SWAN Hyperactivity	191	28.97	6.14	28.00	10, 42	0.13	−0.52
SWAN Impulsivity	191	14.55	3.13	14.00	4, 21	0.03	−0.20
SWAN Total Score	191	85.74	17.03	83.00	29, 121	−0.02	−0.25

*Note. WASI*, Wechsler Abbreviated Scale of Intelligence; *TMT Part B-A Time*, Trail Making Part B minus Part A; *SWAN*, Strengths and Weaknesses of ADHD Symptoms and Normal Behaviors.

**Table 2:** Correlations between age, EF tasks and parent reports of impulse and attention regulation in the full sample.

	Group	1	2	3	4	5	6	7
1. Age	Total Sample	-	−0.63 *	−0.55 *	0.03	0.06	−0.03	−0.04
	ADHD Risk	-	−0.66 *	−0.62 *	−0.12	−0.06	0.04	−0.09
2. Stroop Interference Time	Total Sample		-	0.59 *	−0.18 *	−0.15 *	0.10	−0.16 *
	ADHD Risk		-	0.73 *	−0.18 *	−0.06	−0.14	−0.17
3. TMT Part B-A Time	Total Sample			-	−0.28 *	−0.21 *	−0.16 *	−0.27 *
	ADHD Risk			-	−0.13	−0.03	−0.10	−0.12
4.SWAN Inattention Ratings	Total Sample				-	0.77 *	0.44 *	0.95 *
	ADHD Risk				-	0.47 *	0.30 *	0.88 *
5. SWAN Hyperactivity Ratings	Total Sample					-	0.48 *	0.92 *
	ADHD Risk					-	0.55 *	0.78 *
6. SWAN Impulsivity Ratings	Total Sample						-	0.58 *
	ADHD Risk						-	0.62 *
7. SWAN Total Score Ratings	Total Sample							-
	ADHD Risk							-

*Note.* * *p* < 0.05. Total sample *n* = 337/324; ADHD risk sample *n* = 85. *TMT Part B-A Time*, Trail Making Part B minus Part A; *SWAN*, Strengths and Weaknesses of ADHD Symptoms and Normal Behaviors.

**Table 3:** Correlations between age, EF tasks and parent reports of impulse and attention regulation among 8–13-year-olds.

	Group	1	2	3	4	5	6	7
1. Age	Total Sample	-	−0.25 *	−0.35 *	−0.06	0.01	0.02	−0.02
	ADHD Risk		−0.25	−0.39 *	−0.08	0.02	0.19	0.01
2. Stroop Interference Time	Total Sample		-	0.43 *	−0.26 *	−0.21 **	0.12	−0.23 *
	ADHD Risk		-	0.62 *	−0.42 *	−0.22	−0.27	−0.38 *
3. TMT Part B-A	Total Sample			-	−0.33 *	−0.25 *	−0.22 *	−0.32 *
	ADHD Risk			-	−0.32 *	−0.16	−0.23	−0.29 *
4. SWAN Inattention Ratings	Total Sample				-	0.76 *	0.36 *	0.94 *
	ADHD Risk				-	0.59 *	0.46 *	0.90 *
5. SWAN Hyperactivity Ratings	Total Sample					-	0.39 *	0.92 *
	ADHD Risk					-	0.66 *	0.86 *
6. SWAN Impulsivity Ratings	Total Sample						-	0.51 *
	ADHD Risk						-	0.73 *
7. SWAN Total Score Ratings	Total Sample							-
	ADHD Risk							-

*Note.* * *p* < 0.05. Total sample of 8–13-year-olds *n* = 194/182; ADHD risk sample *n* = 49. *TMT Part B-A Time*, Trail Making Part B minus Part A; *SWAN*, Strengths and Weaknesses of ADHD Symptoms and Normal Behaviors.

**Table 4:** Correlations between age, EF tasks and parent reports of impulse and attention regulation among 14–20-year-olds.

	Group	1	2	3	4	5	6	7
1. Age	Total Sample	-	−0.18 *	−0.16	0.02	0.004	0.01	0.03
	ADHD Risk	-	−0.37 *	−0.38 *	−0.05	−0.30	−0.08	−0.17
2. Stroop Interference Time	Total Sample		-	0.35 *	−0.08	−0.01	−0.04	−0.05
	ADHD Risk		-	0.59 *	−0.15	0.28	0.07	0.01
3. TMT Part B-A	Total Sample			-	−0.28 *	−0.16	−0.18 *	−0.24 *
	ADHD Risk			-	−0.05	0.25	0.19	0.10
4. SWAN Inattention Ratings	Total Sample				-	0.78 *	0.70 *	0.95 *
	ADHD Risk				-	0.32	0.09	0.87 *
5. SWAN Hyperactivity Ratings	Total Sample					-	0.81 *	0.93 *
	ADHD Risk					-	0.40 *	0.71 *
6. SWAN Impulsivity Ratings	Total Sample						-	0.85 *
	ADHD Risk						-	0.45 *
7. SWAN Total Score Ratings	Total Sample							-
	ADHD Risk							-

*Note.* * *p* < 0.05. Total sample of 14–20-year-olds *n* = 143/142; ADHD risk sample *n* = 36. *TMT Part B-A Time*, Trail Making Part B minus Part A; *SWAN*, Strengths and Weaknesses of ADHD Symptoms and Normal Behaviors.

**Table 5:** Rating scales of attention regulation and executive functions: Availability of age-based norms and informant instructions.

Name of Scale	Description	Age Range	Rater	Normed for Age?	Instructions to Rater
Strengths and Weaknesses of Attention-Deficit/Hyperactivity Symptoms and Normal Behaviors (SWAN) rating scale [41,42]	In total, 18 DSM-5 items using competency-based or strength-based descriptions rather than symptoms of ADHD. The items measure behavioral characteristics representative of the attention skills of the general population.	6–18 years	Parent Teacher	No	“Children differ in their abilities to focus attention, control activity, and inhibit impulses. For each item listed below, how does this child compare to other children of the same age? Please select the best rating on your observations over the past month.”
Conners (3rd edition) [66] Short and Full-Length Versions DSM-5 Updated Forms	A multi-informant assessment of children and adolescents that takes into account home, social and school settings. It includes 18 DSM-5 symptoms for ADHD.	6–18 years	Parent Teacher	Yes	“Here, are some things parents might say about their children. Please tell us about ***your*** child and what he/she has been like in the **PAST MONTH**. Read each item carefully, then decide how well it describes your child or how frequently it has happened.”
		8–18 years	Self		
ADHD Rating Scale-5 for Children and Adolescents [67]	Includes 18 DSM-5 item criteria for (ADHD).	Child Version (5–10 years)	Parent Teacher	Yes	“Circle the number that best describes your child’s behavior over the **past 6 months**.”
		Adolescent Version (11–17 years)	Parent Teacher		
NICHQ Vanderbilt ADHD Diagnostic Parent/Teacher Rating Scales (3rd Edition) [68]	Questionnaire used by health care professionals to help diagnose ADHD in children.	6–12 years	Parent Teacher	No	“Each rating should be considered in the context of what is appropriate for the age of your child. When completing this form, please think about your child’s behaviors in the **past 6 months**.”
SNAP-IV ADHD Symptom Checklist (90-item, 18-item and 26-item scales) [69]	Behavior rating scales as assessment tool for diagnosing attention-deficit hyperactivity disorder (ADHD) based on the DSM-IV.	6–18 years	Parent Teacher	No	“For each item, check the column which best describes this child/adolescent: not at all, just a little, quite a bit, or very much.”
Brown Executive Function/Attention Scale (Brown EF/A Scales) [70]	A set of rating scales designed to evaluate executive functions related to attention-deficit/hyperactivity disorder (ADHD).	Primary/ Preschool Version (3–7 years)	Parent Teacher	Yes	“Item by item, read each symptom listed, and circle the number beneath the words that tell how much you believe that feeling or behavior has been a problem for your child in the past 6 months.”
		School-age Version (8–12years)	Parent Teacher Self		
		Adolescent Version (13–18 years)	Parent Self		
Achenbach System of Empirically Based Assessment (CBCL) [71]	Assesses adaptive and maladaptive functioning. Empirically based syndrome scales relevant to ADHD behaviors (attention problems) and DSM-5-oriented scale (attention-deficit/hyperactivity problems).	CBCL (6–18 years)	Parent	Yes	“Below is a list of items that describe children and youths. For each item that describes your child **now or within the past 6 months**, please circle the 2 if the item is very true or often true of your child. Circle the 1 if the item is somewhat or sometimes true of your child. If the item is not true of your child, circle the 0. Please answer all items as well as you can, even if some do not seem to apply to your child.”
		TRF (6–18 years)	Teacher		
		YSR (11–18 years)	Self		
Behavior Assessment System for Children—Third Edition (BASC-3) [72]	A comprehensive assessment of behavior and emotions for children and adolescents. Scales such as hyperactivity and attention problems are relevant behavioral ratings for ADHD.	2–21 years	Parent (PRS) Teacher (TRS)	Yes	“This form contains phrases that describe how children may act. Please read each phrase and select the response that describes how this child has behaved recently (in the last several months).”
		6 years through college age	Self		
The Strengths and Difficulties Questionnaire (SDQ) [73]	A brief behavioral screening questionnaire for children and adolescents. It exists in several versions to meet the needs of researchers, clinicians and educationalists. All versions of the SDQ ask about 25 attributes, some positive and others negative.	Preschool 2–4 years	Parent/ Teacher	No	“For each item, please mark the box for Not True, Somewhat True or Certainly True. It would help us if you answered all items as best you can even if you are not absolutely certain or the item seems daft! Please give your answers on the basis of the child’s behavior over the last six months.”
		School-age 4–17 years	Parent Teacher		
		11–17 years	Self		
Clinical Assessment of Attention Deficit—Child (CAT-C) [74]	A questionnaire that provides a comprehensive assessment of attention deficit disorder with and without hyperactivity. Linkage to the DSM-IV diagnostic criteria, with comprehensive content coverage both within and across scales/clusters assists in rendering a differential diagnosis.	8–18 years	Parent Teacher Self	Yes	“Please read these instructions before completing this Rating Form. Mark all of your answers directly on this form. This booklet has sentences that may describe your **CHILD** lately. Please read each sentence carefully and select the response that best describes how much you agree or disagree with each sentence. Then, circle the number that matches your answer. Circle one response for each sentence.”
Behavior Rating Inventory of Executive Function (BRIEF-2), Second Edition [75] Screening and Full-Length Versions	The BRIEF-2 is a rating scale that assesses executive function in the children and adolescents. It is designed to assist school psychologists as they assess, plan interventions for and monitor students with executive dysfunction.	5–18 years	Parent Teacher	Yes	“Below is a list of statements that describe children. We would like to know if your child has had problems with these behaviors over the past 6 months. Please answer all the items the best that you can. Please DO NOT SKIP ANY ITEMS. Think about your child as you read each statement and circle.”
		11–18 years	Self		
The Childhood Executive Functioning Inventory (CHEXI) [76] Teenage Executive Functioning Inventory (TEXI) [77]	A rating of instruments for parents and teachers that was developed in 2008 for measuring executive function.	4–12 years	Parent or Teacher	No	“Below, you will find a number of statements. Please read each statement carefully and thereafter indicate how well that statement is true for the child. You indicate your response by circling one of the numbers (from 1 to 5) after each statement.”
		13–19 years	Parent/ Teacher Self		
Barkley Deficits in Executive Functioning Scale: Children and Adolescents (BDEFS-CA) [78]	The Appendix contains long forms (10–15 min) and short forms (3–5 min) for parents to complete and profiles. A short clinical interview form based on the short-form rating scale, for use in unusual circumstances where a parent is unable to complete a rating scale. It is an empirically based tool for evaluating clinically significant dimensions of child and adolescent executive functioning (EF).	6–17 years	Parent	Yes	“How often does your child experience each of these problems? Please circle the number next to each item that best describes his/her behavior **DURING THE PAST 6 MONTHS**. If your child is currently taking medication for any psychiatric or psychological disorder, please rate his/her behavior based on how he/she acts while **OFF the medication**.”
Delis Rating of Executive Functions (D-REF) [79]	A behavior rating scale designed to assess behaviors that may reflect difficulties with executive functioning.	5–18 years	Parent Teacher	Yes	“The following statements describe behaviors and feelings of children and adolescents. Please read each statement carefully and decide which frequency applies to your child within the past 6 months. Remember to give your own opinion of the child’s behavior and select the frequency that you feel best applies. If you change your mind, mark through the answer you want to change and circle the new one: Circle S/N for Seldom/Never if the behavior occurs less than once every 3 months or never. Circle M for Monthly if the behavior occurs at least once every 1–3 months (and less than once a week). Circle W for Weekly if the behavior occurs at least once a week (and less than once a day). Circle D for Daily if the behavior occurs at least once a day”
		11–18 years	Self		

* Disclaimer: not an exhaustive list.

## Data Availability

We do not have consent from participants to make data publicly available or to post data on an online repository.
